# Vaccinations and Dravet Syndrome

**DOI:** 10.15844/pedneurbriefs-29-11-4

**Published:** 2015-11

**Authors:** Christian M. Korff

**Affiliations:** 1 Pediatric Neurology, Department of Pediatrics, University Hospitals of Geneva, Geneva, Switzerland

**Keywords:** Dravet Syndrome, Vaccinations, Prognosis, Seizure Risk, Development

## Abstract

Investigators from various university hospitals, reference medical institutions and epilepsy centers, and the national institute for public health and environment in the Netherlands, studied the effect of vaccinations on seizure risk and disease course in patients with Dravet syndrome (DS).

Investigators from various university hospitals, reference medical institutions and epilepsy centers, and the national institute for public health and environment in the Netherlands, studied the effect of vaccinations on seizure risk and disease course in patients with Dravet syndrome (DS). They retrospectively estimated the risk of subsequent seizures after infant whole-cell pertussis (wP), acellular pertussis (aP), and nonpertussis (non-P) combination vaccination, and measles-mumps-rubella (MMR) vaccination in a nationwide cohort of 77 children with DS and pathogenic SCN1A mutations. They analyzed the influence of age at seizure-onset and age at appearance of developmental delay, and compared their results in two subgroups of patients: those with and those without vaccination-associated seizure onset (defined as seizures appearing within 24 hours after administration of an inactivated (pertussis combination) vaccine or within 5 to 12 days after administration of a live attenuated MMR vaccine).

Their results show that children with vaccination-associated seizure onset were significantly younger at first seizure than those without vaccination-associated seizure onset, but age at first seizure unrelated to vaccination, and age at first report of developmental delay or cognitive outcome did not differ between both groups. In addition, the risk of subsequent vaccination-associated seizures was significantly lower for aP and non-P than for wP, and there was an increased incidence rate ratio of seizures of 2.3 following MMR vaccination. [1]

COMMENTARY. This study shows that patients with DS who enter their disease with vaccination-associated seizures do not have a different overall prognosis than those with initial seizures unrelated to vaccinations. In addition, although the absolute risk of seizure after various vaccinations is substantial in DS, the risk of subsequent vaccination-associated seizures is likely to be vaccine-specific, and mainly concerns MMR immunization (although this did not differ from the relative seizure risk in the general pediatric population). These results confirm that a diagnosis of DS is not a contraindication for vaccinations, even in children who presented seizures related to vaccinations at onset. As stated by the authors, it remains nevertheless important to try and select vaccines that carry lower risks of seizures in these children who are particularly prone to develop prolonged seizures, in order to prevent the potential acute encephalopathy described in certain patients with DS after status epilepticus [2].
